# Supplementary material for: Identification of factors directly linked to incident chronic obstructive pulmonary disease: A causal graph modeling study
Source: PLoS Med. 2024 Aug 13;21(8):e1004444. doi: 10.1371/journal.pmed.1004444 (PMC11349214; doi:10.1371/journal.pmed.1004444)
Supplement: S1 Fig — (A) The progression of GOLD stage is visualized for baseline (visit 1), 5-year follow-up (visit 2), and 10-year follow up (visit 3). Individuals with a missing GOLD stage status were assigned to the “Unknown” category. A total of 2,643 GOLD 0 individuals had known GOLD Stage statuses between the first and second visit while 471 GOLD 0 individuals had known GOLD Stage statuses between the second and third visit. (B) Shows the breakdown of these individuals as they transition between visits. A Chi-squared test revealed no significant difference between visit transitions (p = 0.21). GOLD, Global Initiative for Obstructive Lung Disease. (PDF) [file pmed.1004444.s002.pdf]

A

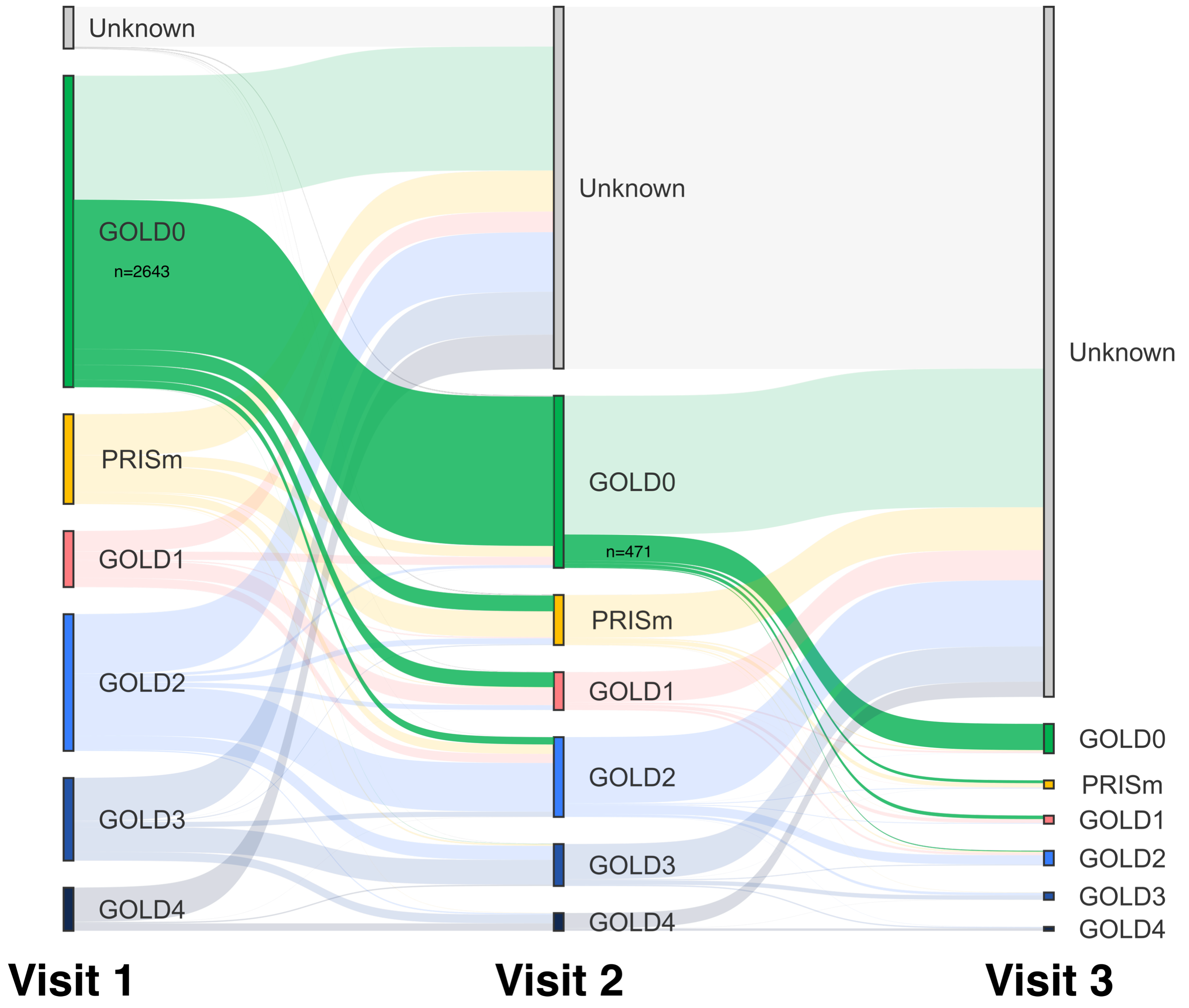

B

GOLD 0 Subjects Transitioning to next Visit

New GOLD Stage Status	Visit 1 → Visit 2	Visit 2 → Visit 3
GOLD 0	2105 (79.6%)	369 (78.3%)
GOLD 1	210 (7.95%)	50 (10.6%)
GOLD 2	103 (3.90%)	12 (2.55%)
GOLD 3	2 (0.08%)	0 (0.00%)
GOLD 4	0 (0.00%)	0 (0.00%)
PRISm	223 (8.44%)	40 (8.49%)
Total	2643	471

Chi-Squared Test, p=0.21

S1 Figure. GOLD Stage transitions across all time points. (A) The progression of GOLD stage is visualized for baseline (visit 1), 5-year follow-up (visit 2), and 10-year follow up (visit 3). Individuals with a missing GOLD stage status were assigned to the “Unknown” category. 2643 GOLD 0 individuals had known GOLD Stage statuses between the first and second visit while 471 GOLD 0 individuals had known GOLD Stage statuses between the second and third visit. (B) Shows the breakdown of these individuals as they transition between visits. A Chi-squared test revealed no significant difference between visit transitions (p=0.21). **Abbreviations:** GOLD: Global Initiative for Obstructive Lung Disease.
