# Supplementary material for: Identification of factors directly linked to incident chronic obstructive pulmonary disease: A causal graph modeling study
Source: PLoS Med. 2024 Aug 13;21(8):e1004444. doi: 10.1371/journal.pmed.1004444 (PMC11349214; doi:10.1371/journal.pmed.1004444)
Supplement: S2 Fig — From the 623 baseline variables, variables were removed for having information about future visits, being duplicate/constant/uninformative, having too many missing values, having categories that were too small, or for being too correlated. (PDF) [file pmed.1004444.s003.pdf]

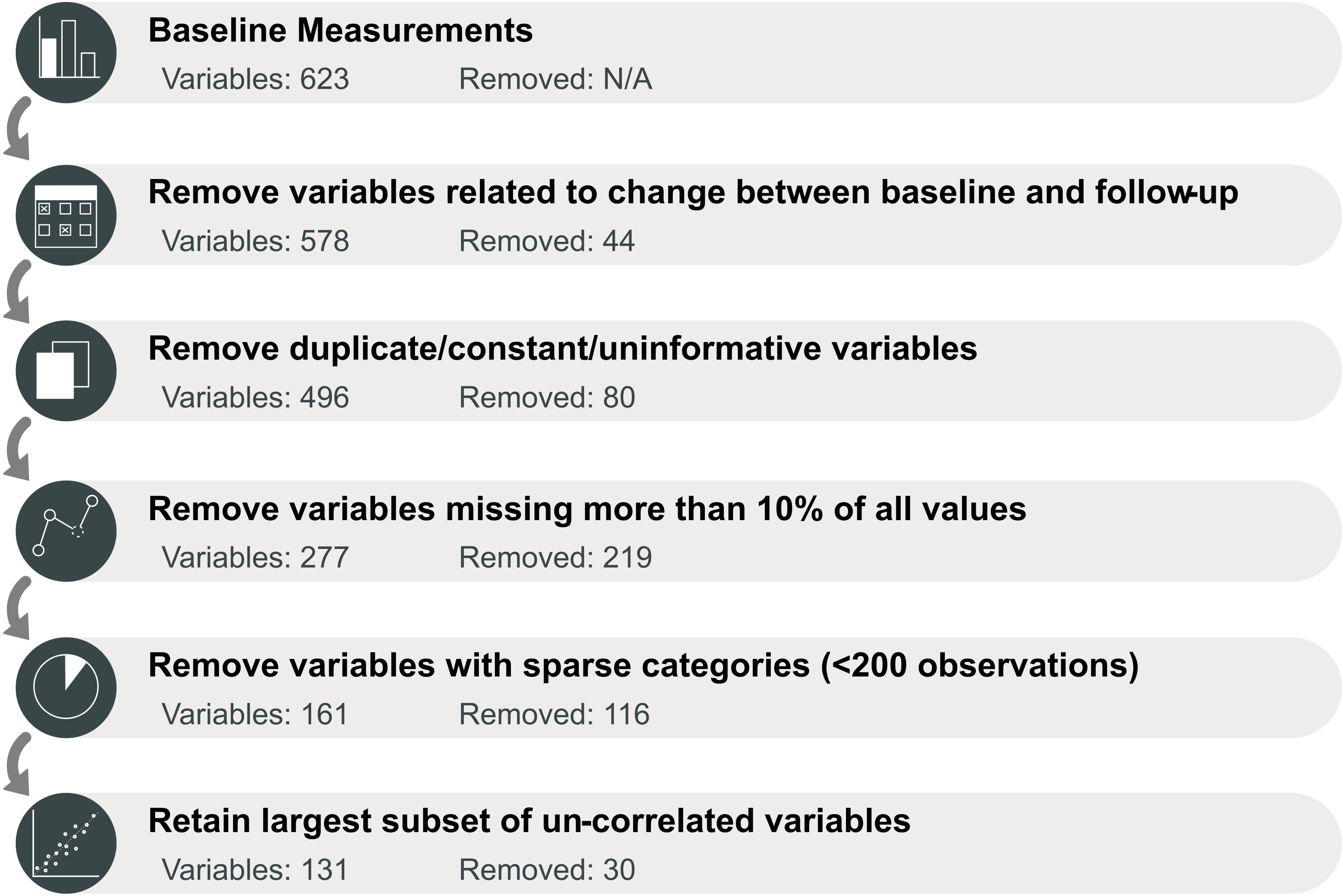

S2 Figure. Procedure to filter baseline variables. From the 623 baseline variables, variables were removed for having information about future visits, being duplicate/constant/uninformative, having too many missing values, having categories that were too small, or for being too correlated.
