# Supplementary material for: Identification of factors directly linked to incident chronic obstructive pulmonary disease: A causal graph modeling study
Source: PLoS Med. 2024 Aug 13;21(8):e1004444. doi: 10.1371/journal.pmed.1004444 (PMC11349214; doi:10.1371/journal.pmed.1004444)
Supplement: S3 Fig — For each data division, the distribution of age is plotted along with the mean (black circle) and standard deviation (vertical line). Mann–Whitney–Wilcoxon tests were performed for each pairwise comparison. Age was significantly different in the validation data set for each comparison. A Kruskal–Wallis test was also performed and showed global significance across data splits. (PDF) [file pmed.1004444.s004.pdf]

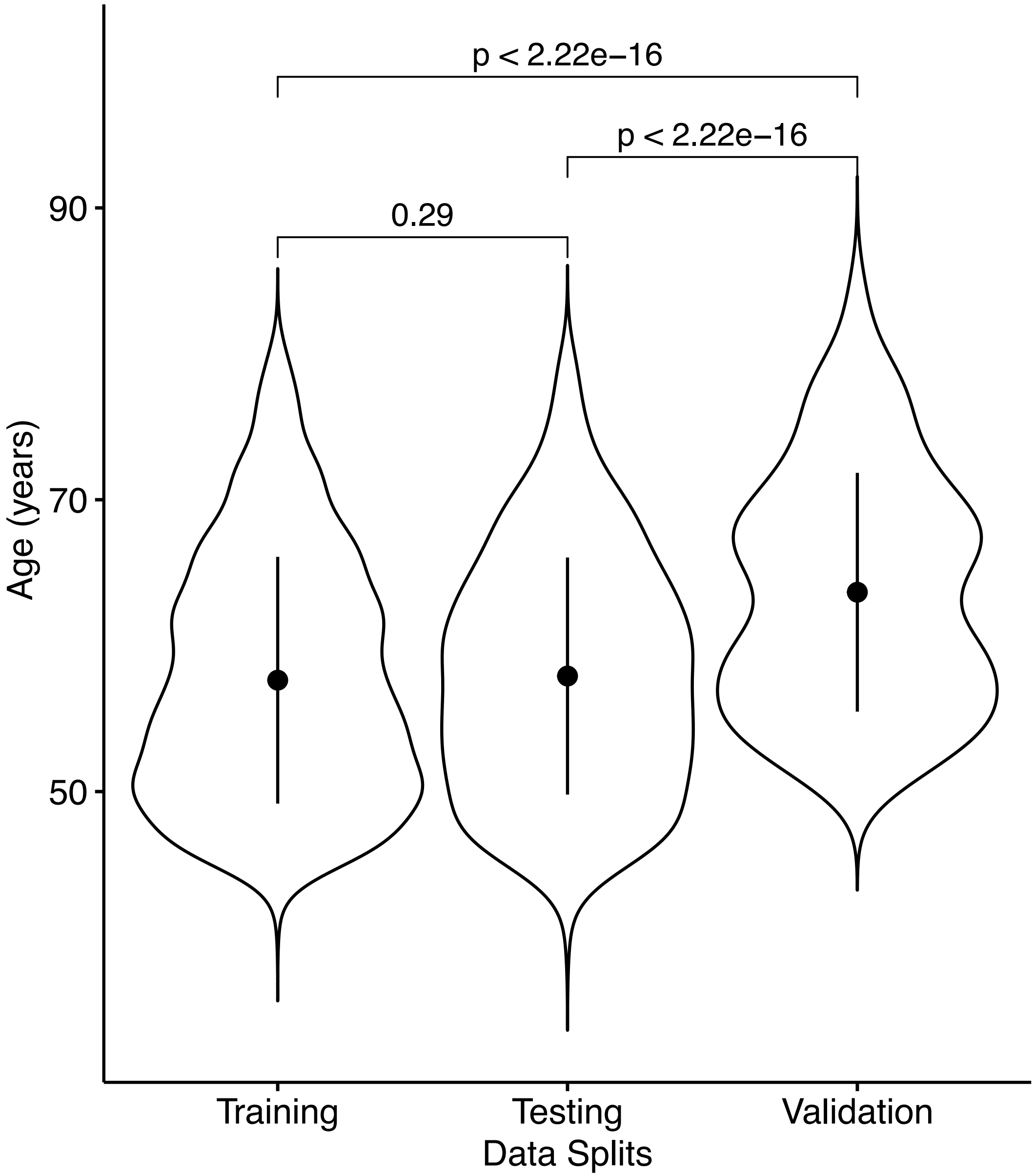

S3 Figure. Age distribution across data splits. For each data division, the distribution of age is plotted along with the mean (black circle) and standard deviation (vertical line). Mann-Whitney-Wilcoxon tests were performed for each pairwise comparison. Age was significantly different in the validation dataset for each comparison. A Kruskal-Wallis test was also performed and showed global significance across data splits.
