# Supplementary material for: Identification of factors directly linked to incident chronic obstructive pulmonary disease: A causal graph modeling study
Source: PLoS Med. 2024 Aug 13;21(8):e1004444. doi: 10.1371/journal.pmed.1004444 (PMC11349214; doi:10.1371/journal.pmed.1004444)
Supplement: S4 Fig — Each edge in the graph is labeled with the number of times it appeared in a Markov blanket across each fold and graph sparsity iteration (10 folds and 10 sparsities). (A) Shows every Markov blanket variable when only FEV1 and FVC variables were removed. (B) Shows only first neighbors to with no spirometry variables. CT, computed tomography; Pi10, average lung wall thickness in 10 mm radius; FEF25-75%, forced expiratory flow in the middle range; PEF, peak expiratory flow. (PDF) [file pmed.1004444.s005.pdf]

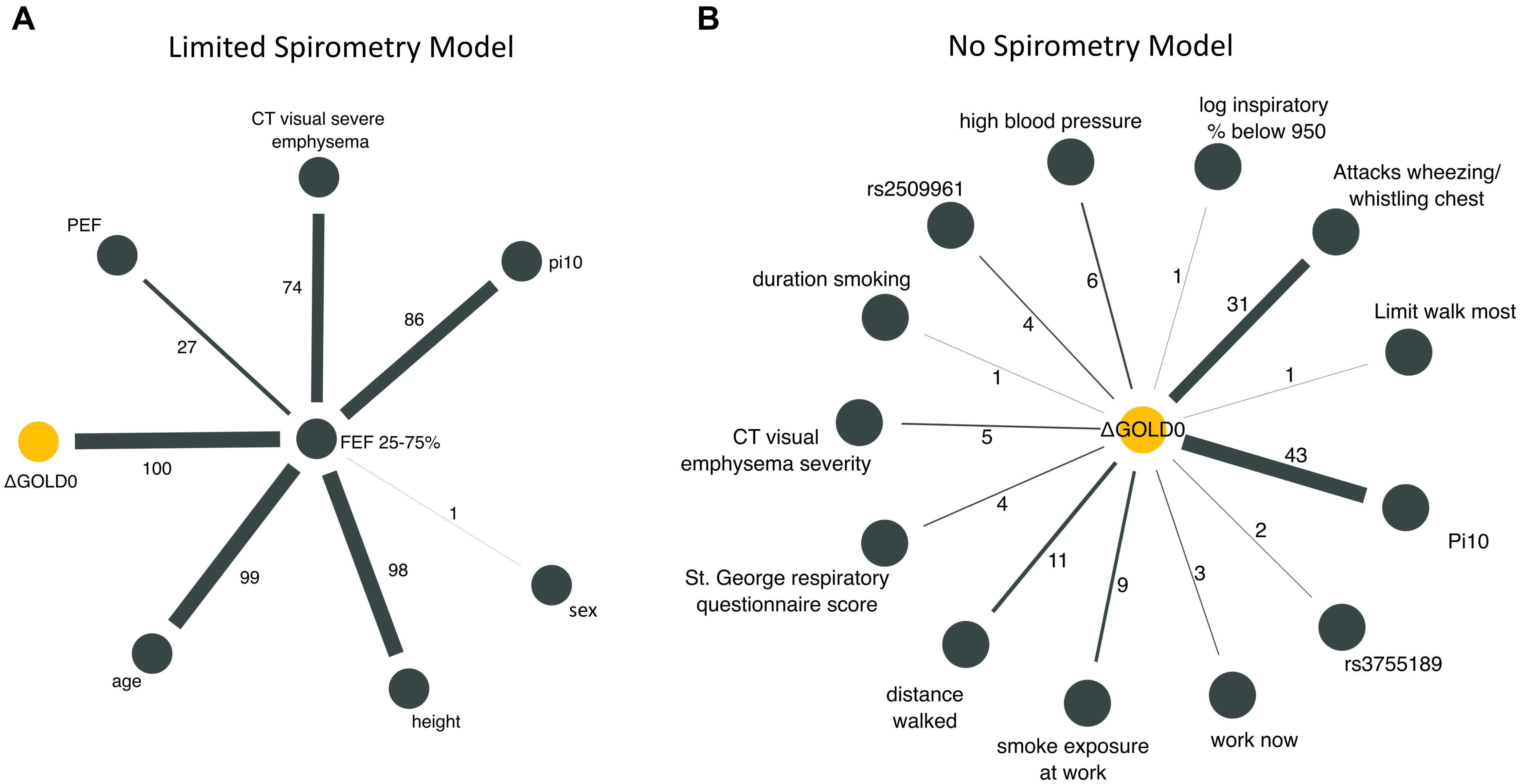

S4 Figure. Markov Blanket variable counts. Each edge in the graph is labeled with the number of times it appeared in a Markov blanket across each fold and graph sparsity iteration (10 folds and 10 sparsities). (A) Shows every Markov blanket variable when only FEV1 and FVC variables were removed. (B) Shows only first neighbors to with no spirometry variables. **Abbreviations:** CT, computed tomography; Pi10: average lung wall thickness in 10 mm radius; FEF25-75%, forced expiratory flow in the middle range; PEF: peak expiratory flow.
