# Supplementary material for: Identification of factors directly linked to incident chronic obstructive pulmonary disease: A causal graph modeling study
Source: PLoS Med. 2024 Aug 13;21(8):e1004444. doi: 10.1371/journal.pmed.1004444 (PMC11349214; doi:10.1371/journal.pmed.1004444)
Supplement: S5 Fig — Receiver operator curves illustrate model performance for FCI-Max + Logistic Regression and random forest. Area under the curve measurements are displayed in the legend. (A–C) Include “limited spirometry” models. (D–F) Includes “no spirometry” models. AUROC, area under the receiver operator characteristic curve. (PDF) [file pmed.1004444.s006.pdf]

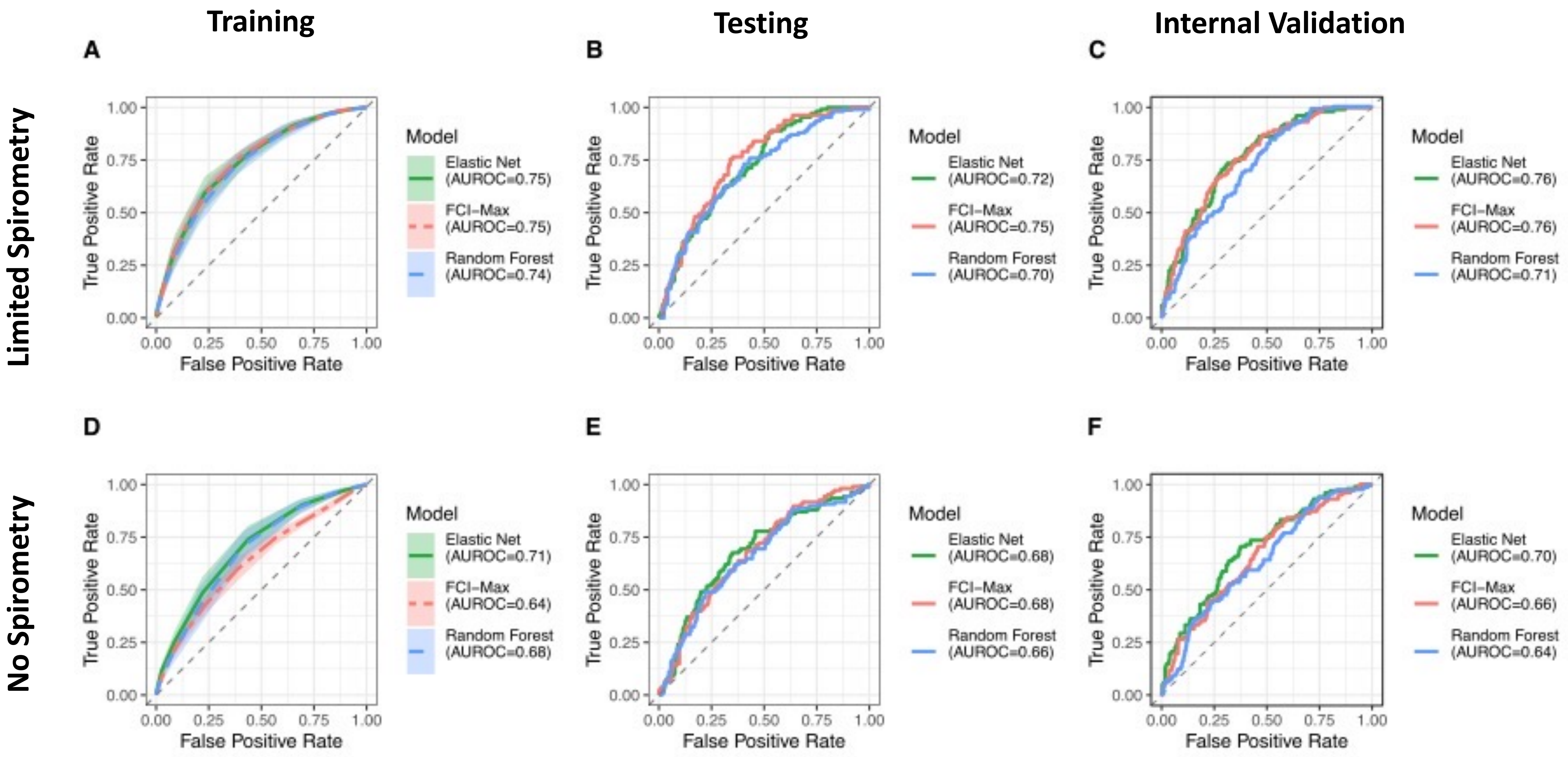

S5 Figure. Classifier performance for predicting change in GOLD 0 status. Receiver Operator Curves illustrate model performance for FCI-Max + Logistic Regression and Random Forest. Area under the curve measurements are displayed in the legend. (A-C) Include "limited spirometry" models (D-F) Includes "no spirometry" models. **Abbreviations:** AUROC, area under the receiver-operator characteristic curve.
