# Supplementary material for: Identification of factors directly linked to incident chronic obstructive pulmonary disease: A causal graph modeling study
Source: PLoS Med. 2024 Aug 13;21(8):e1004444. doi: 10.1371/journal.pmed.1004444 (PMC11349214; doi:10.1371/journal.pmed.1004444)
Supplement: S6 Fig — (A) COPDGene cohort, (B) SCCOR cohort. SCCOR has lower median and variability of FEF25-75% and higher separation of those individuals that leave GOLD 0 (i.e., develop lung abnormalities). GOLD, Global Initiative for Obstructive Lung Disease; FEF25-75%, forced expiratory flow in the middle range. (PDF) [file pmed.1004444.s007.pdf]

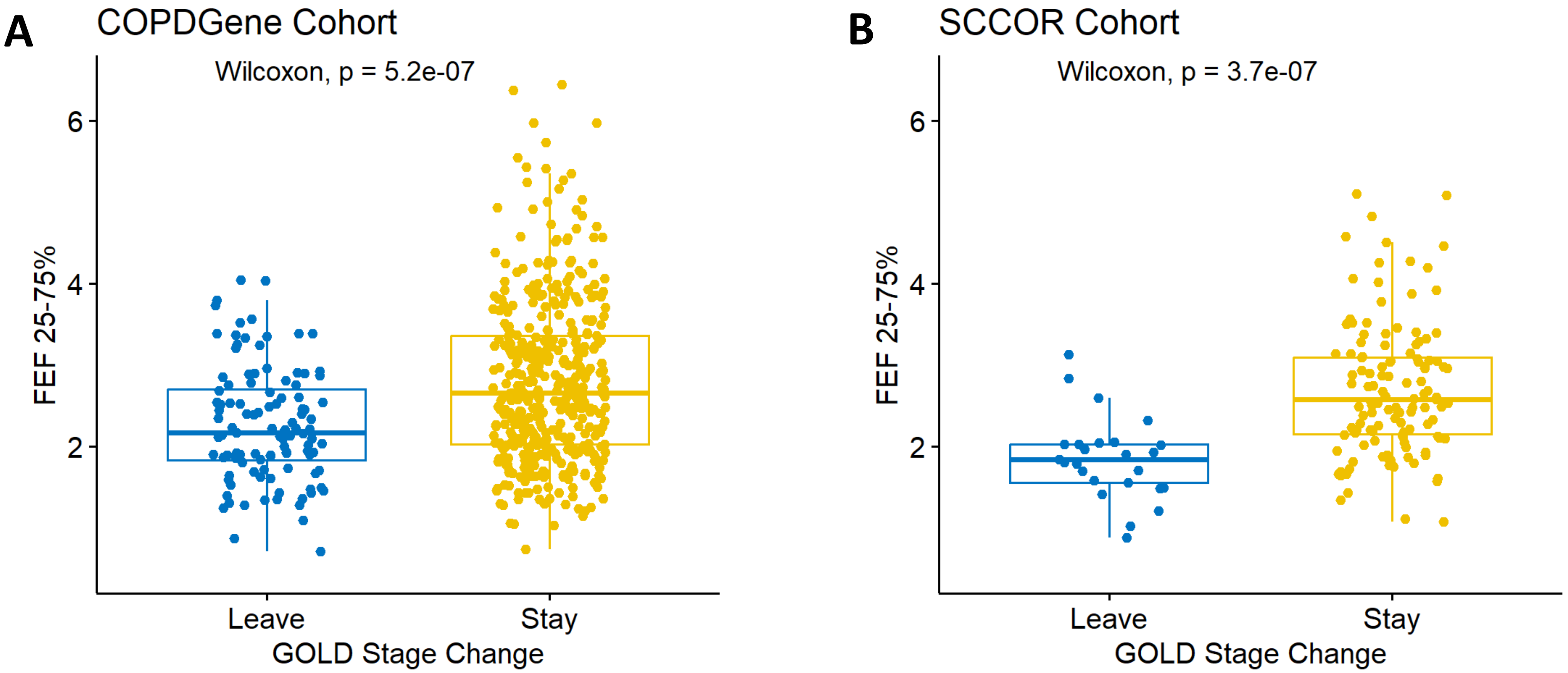

S6 Figure. Boxplots indicating the separation with respect to the $FEF_{25\text{-}75\%}$ of the two classes (leave, stay in GOLD 0). **(A)** COPDGene cohort, **(B)** SCCOR cohort. SCCOR has lower variability and higher separation of the two outcomes than COPDGene. **Abbreviations:** GOLD: Global Initiative for Obstructive Lung Disease; FEF25-75%, forced expiratory flow in the middle range.
