# Supplementary material for: Identification of factors directly linked to incident chronic obstructive pulmonary disease: A causal graph modeling study
Source: PLoS Med. 2024 Aug 13;21(8):e1004444. doi: 10.1371/journal.pmed.1004444 (PMC11349214; doi:10.1371/journal.pmed.1004444)
Supplement: S7 Fig — (A, B) The 2 model prediction results with all 471 individuals who were GOLD 0 status at the 5-year follow-up and had a known GOLD stage status at the 10-year follow-up. This is the internal validation data set. (C, D) The same results but on a subset of 77 five-year follow-up GOLD 0 status individuals who were part of the initial testing data set. These individuals were not used at all during training. AUROC, area under the receiver operator characteristic curve. (PDF) [file pmed.1004444.s008.pdf]

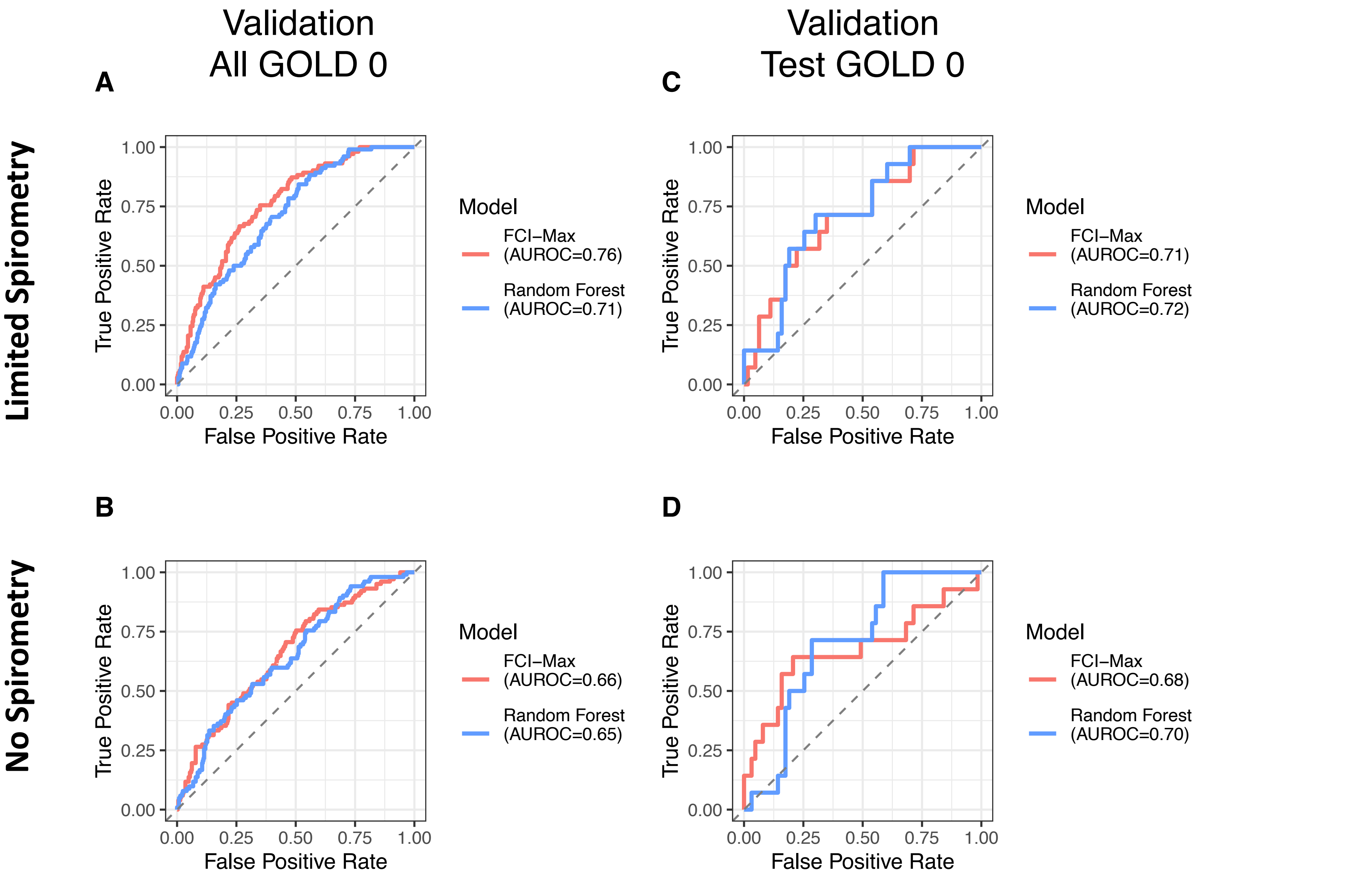


**S7 Figure.** Comparing ΔGOLD0 predictions for different validation datasets. (A-B) Show model prediction results with all 471 subjects who were GOLD 0 status at the 5-year follow-up and had a known GOLD stage status at the 10-year follow-up. (C-D) Show the same results but on a subset of 77 5-year follow-up GOLD 0 status subjects. Specifically, only subjects from the testing dataset are used (as opposed to the training and testing datasets). **Abbreviations:** AUROC, area under the receiver-operator characteristic curve.
