# Supplementary material for: Identification of factors directly linked to incident chronic obstructive pulmonary disease: A causal graph modeling study
Source: PLoS Med. 2024 Aug 13;21(8):e1004444. doi: 10.1371/journal.pmed.1004444 (PMC11349214; doi:10.1371/journal.pmed.1004444)
Supplement: S8 Fig — (A) GOLD 0 COPDGene participants that develop spirometric abnormalities at 5-year follow-up visit (“Leave GOLD 0”; red dots) and those that remain GOLD 0 (gray dots). (B) Correct prediction of those “Leaving GOLD 0” by the limited spirometry model (blue dots) and misclassified individuals (orange dots). GOLD, Global Initiative for Obstructive Lung Disease; FEV1, forced expiratory volume in 1 s; FVC, forced vital capacity. (PDF) [file pmed.1004444.s009.pdf]

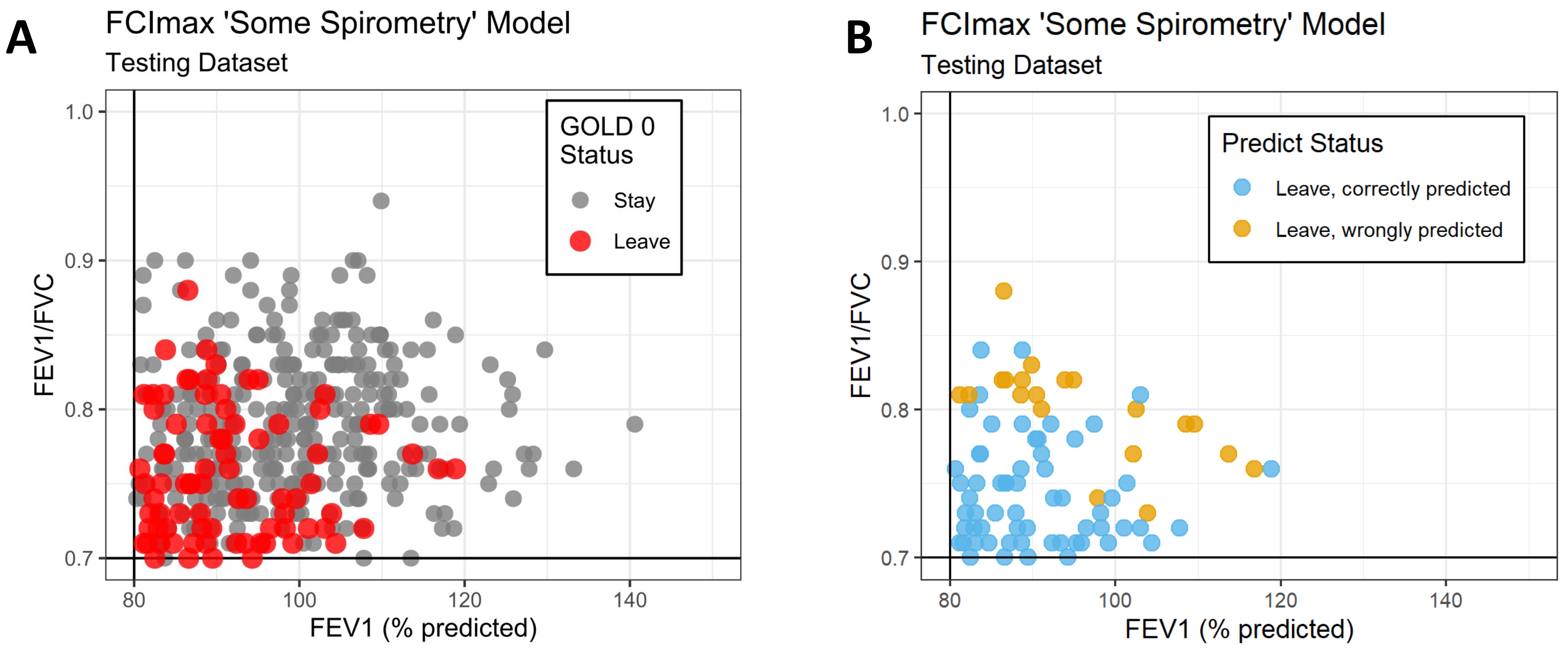

S8 Figure. (A) GOLD 0 COPDGene subjects that develop spirometric abnormalities at 5-yr follow up visit ("Leave GOLD 0"; red dots) and those that remain GOLD 0 (grey dots). **(B)** Correct prediction of those "Leaving GOLD 0" by the *limited spiromtery* model (blue dots) and misclassified subjects (orange dots). **Abbreviations:** GOLD: Global Initiative for Obstructive Lung Disease; FEV1, forced expiratory volume in one second; FVC, forced vital capacity.
