# Supplementary material for: Identification of factors directly linked to incident chronic obstructive pulmonary disease: A causal graph modeling study
Source: PLoS Med. 2024 Aug 13;21(8):e1004444. doi: 10.1371/journal.pmed.1004444 (PMC11349214; doi:10.1371/journal.pmed.1004444)
Supplement: S9 Fig — (A) Variables are ordered by importance using mean absolute Shapley values. (B–D) Show the distribution of Shapley values across measured demographics (age, biological sex, and race). Positive Shapley values on vertical axes indicate the random forest model was more likely to predict that individual to leave the GOLD 0 status (and vice versa). In (B) we do not display 4 (out of 529) individuals that were <45 years old at baseline, since they did not match the inclusion criteria of COPDGene. (E) Describes the contributions SNPs had to the random forest model prediction. Colors differentiate individuals with and without a given SNP. Pi10, average lung wall thickness in 10 mm radius; HU, Hounsfield units; SGRQ, St George Respiratory Questionnaire. (PDF) [file pmed.1004444.s010.pdf]

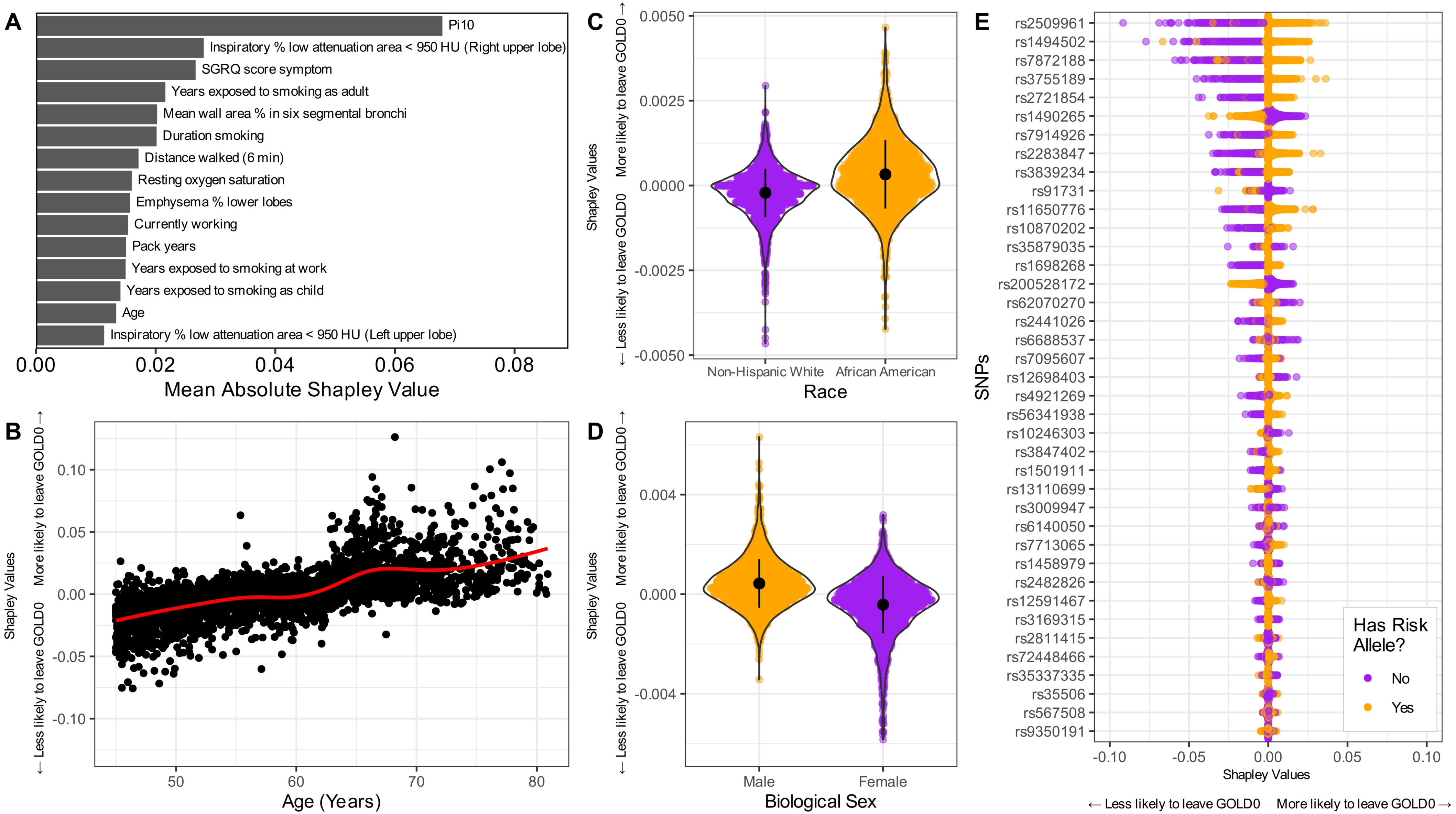

S9 Figure. Exploring variable important within the Random Forest Model ("no spirometry" model). **(A)** Variables are ordered by importance using mean absolute Shapley values. **(B-D)** Show the distribution of Shapley values across measured demographics (age, biological sex, and race). Positive Shapley values on vertical axes indicate the random forest model was more likely to predict that individual to leave the GOLD 0 status (and vise vera). In (B) we do not display 4 (out of 529) individuals that were <45 yrs old at baseline, since they did not match the inclusion criteria of COPDGene. **(E)** Describes the contributions SNPs had to the random forest model prediction. Colors differentiate individuals with and without a given SNP. **Abbreviations:** Pi10: average lung wall thickness in 10 mm radius; HU: Hounsfield Units; SGRQ: St George Respiratory Questionnaire.
