# Supplementary material for: Identification of factors directly linked to incident chronic obstructive pulmonary disease: A causal graph modeling study
Source: PLoS Med. 2024 Aug 13;21(8):e1004444. doi: 10.1371/journal.pmed.1004444 (PMC11349214; doi:10.1371/journal.pmed.1004444)
Supplement: S10 Fig — Plotted are the averaged cross fold validated ROC plots. Each legend entry provides the area under the receiver operator curve (AUROC). The dashed gray line represents an uninformative model. AUROC, area under the receiver operator characteristic curve. (PDF) [file pmed.1004444.s011.pdf]

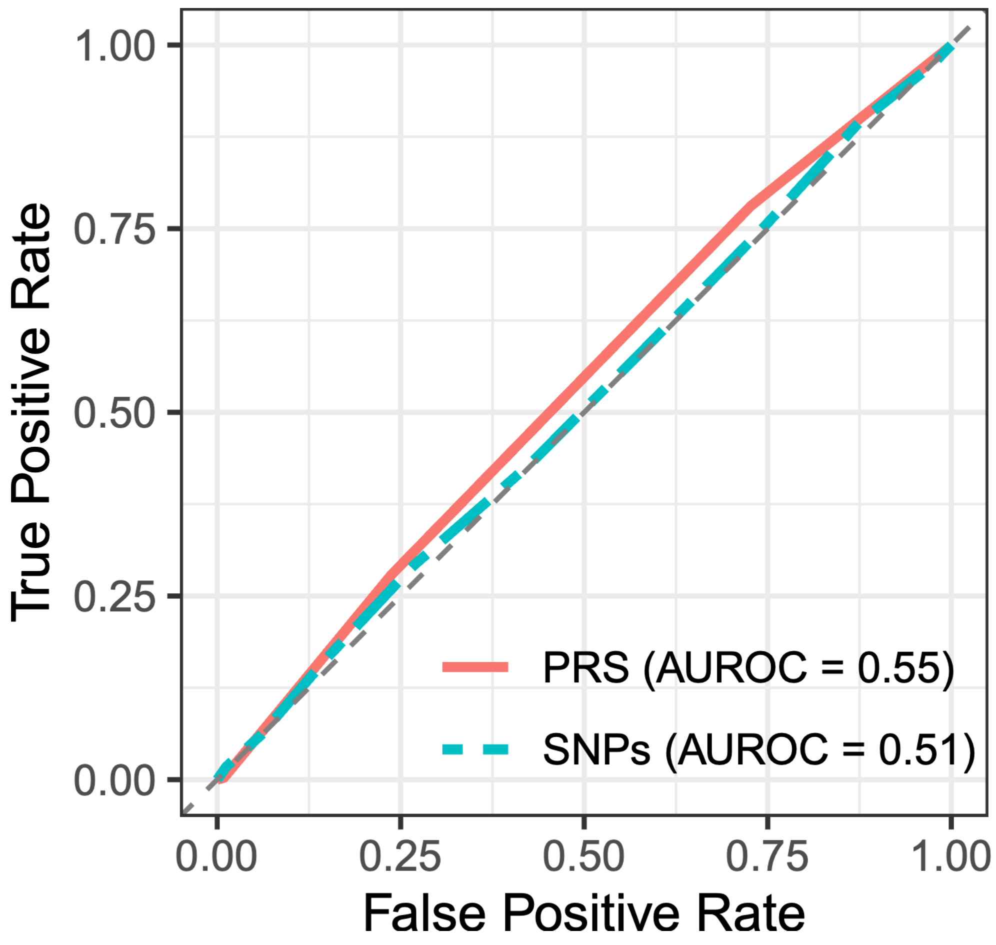

S10 Figure. Polygenic Risk Scores (PRS) outperform SNPs in classifying ΔGOLD0. Plotted are the averaged cross fold validated ROC plots. Each legend entry provides the area under the receiver operator curve (AUROC). The dashed grey line represents an uninformative model. **Abbreviations:** AUROC, area under the receiver-operator characteristic curve.
