# Supplementary material for: Identification of factors directly linked to incident chronic obstructive pulmonary disease: A causal graph modeling study
Source: PLoS Med. 2024 Aug 13;21(8):e1004444. doi: 10.1371/journal.pmed.1004444 (PMC11349214; doi:10.1371/journal.pmed.1004444)
Supplement: S1 Materials — Details are provided for the nested cross-validation scheme and the elastic net regression method. (DOCX) [file pmed.1004444.s012.docx]

**SUPPLEMENTAL MATERIALS and METHODS**

**Nested Cross Validation**

To simultaneously tune hyperparameters and fit predictive models, nested cross validation was performed using the *tidymodels* R package (v1.1.1). After the initial 80/20 training/testing split, the training data was further subdivided into ten cross-folds stratified by change in GOLD0 status. This is to ensure each fold of the data has approximately the same percentage of subject staying and leaving GOLD0. Models are trained on nine of cross folds (called the analysis set) and performance is checked on the remaining cross fold (called the assessment set). Latin hypercube sampling is used to generate 100 possible hyperparameter combinations for the random forest models (see parameters below) and ten logarithmically spaced alpha values are chosen for the causal discovery/logistic regression model (between 10^-5^ and 10^-1^). For each candidate parameter set, the model is trained and assessed on each of the cross folds to predict if an individual subject will leave or stay in the GOLD0 status. This leads to a large number of models (one for each fold and hyperparameter set combination). The best model is chosen by maximizing the AUROC. Finally, to determine how generalizable our selected model is, we predict GOLD0 classification for the left out testing dataset which was not used for any training purposes.

| **Hyperparameter** | **Range** | **Description** |
| --- | --- | --- |
| mtry | [1, 100] | Number of variables to sample for each split in tree |
| trees | [1, 2000] | Number of trees in random forest |
| min_n | [2, 40] | Minimum number of samples within the terminal nodes |

**Elastic Net Regression**

A common method to perform feature selection for linear models is LASSO (least absolute shrinkage and selection operator) regression. Similar to linear regression, LASSO attempts to choose coefficients for the predictors to minimize the residual sum of squares between predicted and estimated outcomes. LASSO differs by adding a term to minimize the absolute sum of coefficients (L1 norm) which shrinks the least associated coefficients to zero, effectively removing them from the model. Here we attempt to predict change in GOLD0 status using Elastic Net regression which combines LASSO with ridge regression (another regularization method that minimizes the L2 norm to mitigate multicollinearity). Here we use the *tidymodels* R package (v1.1.1) in conjunction with the *glmnet* package (v4.1.8) to fit an elastic net model. The same nested cross validation scheme described above is used to determine hyperparameters (called lambda values that control the strength of regularization for LASSO and Ridge). Model predictions are compared with FCImax and random forest models for training, testing, and internal validation in Supplemental Figure 5.

This study is reported as per the Transparent Reporting of a Multivariable Prediction Model for Individual Prognosis Or Diagnosis (TRIPOD) statement (S9 Checklist).
