# Supplementary material for: Identification of factors directly linked to incident chronic obstructive pulmonary disease: A causal graph modeling study
Source: PLoS Med. 2024 Aug 13;21(8):e1004444. doi: 10.1371/journal.pmed.1004444 (PMC11349214; doi:10.1371/journal.pmed.1004444)
Supplement: Attachment — Submitted filename: 2_COPD_onset.Response_A1.0240530-pvb.pdf [file pmed.1004444.s013.pdf]

RESPONSE TO REVIEWERS

We would like to thank the reviewers for the time they devoted to do a thorough review of our paper and the feedback they provided, which helped us improve our paper. Below, we include a point-by-point response to all of their comments.

Reviewer #1:

"Causal Graph Modeling Identifies Six Factors Linked to Incident COPD" describes the development of a linear (graph-based) logistic regression (LR) model using six input variables, for predicting the progression of chronic obstructive pulmonary disease (COPD) over five/ten years. In particular, the graph-based causal modelling to identify six key variables was found to perform similarly to random forest (RF) models utilizing all 131 variables on both a COPDGene validation dataset and an external SCCOR dataset, which was claimed to indicate the lack of significant non-linear associations, and also the viability of implementing a simple regression model for COPD progression. The reduction in number of required variables would allow the model to be more accessible, and possibly generalizable (due to reduced chance of overfitting).

While the determination of causality amongst variables in COPD progression is well-motivated, some issues might be considered:

1. In the Abstract and Introduction, metrics such as "FEV1" and "FVC" are introduced without explaining their significance. Such metrics & abbreviations might be explained at first occurrence.

[Response] We thank the reviewer for pointing this out. We included the definitions of these two variables in the Introduction as they are important for understanding the manuscript. Their significance and utility are further emphasized in the Introduction as the determinants of COPD severity.

2. In the Introduction, it is claimed that "Some methods, like LASSO regression, compensate through sparsity constraints, but this simply identifies variables correlated to outcomes as opposed to potential causal relationships". However, it is not empirically established that variable correlation is inferior to causal determination, in the study that follows. Since the shortcoming of LASSO regression/correlation is claimed, such methods might be included in the experiments (together with RF).

[Response] The reviewer is right that the correlates identified by LASSO are not generally inferior in terms of predictability of target variables. On the other hand, empirical studies from our group and others have shown that, in most cases, causal graphs identify fewer but equally informative features compared to LASSO. An additional advantage of the causal graph variable selection is that the variables it selects are usually more stable (in a cross-validation sense) and represent more direct connections. Having said that, we followed the reviewer's suggestion, and we are now including the results of an Elastic Net regression (a generalization of LASSO) in the Supplementary Materials.

3. Related to the above, on the main claim that logistic regression models perform similarly to RF (and thus no significant non-linear associations for the COPD progression task), the most relevant comparison between LR with 6 input variables and RF with 6 input variables, appears not to have been attempted (the comparison was against RF with 131 input variables). This might be included to confirm the non-inferiority of LR given identical inputs.

[Response] This is an interesting point. The reason we did not try RF with the selected 6 variables is that the 6 variables were picked using the (linear) causal graph. By supplying the RF with all variables, we allow it to choose any non-linear interactions even outside this set of 6 variables. Both algorithms were given identical inputs (the 131 variables), but FCImax + logistic regression identified these 6 variables as the only relevant ones for prediction.

Typically, tuning hyperparameters for RF models circumvents reduction in accuracy by including uninformative features (Probst et al., 2019). But to be sure, and following the reviewer's suggestion, we re-ran the RF model with just the six predictors. The AUROC curve in the training set (average of cross-validations) was 0.674, a decrease from 0.74; and in the testing data 0.708 (same as before). Below we show the respective curves.

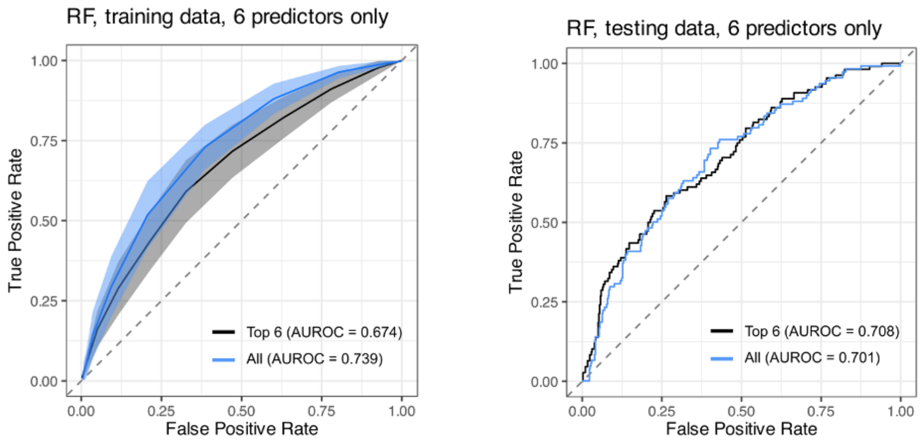

4. In the COPDGene Study Population subsection, there appears a significant proportion of dropouts (i.e. transition to "Unknown" status, in Supplemental Figure S1) over 5 and 10 year visits. Potential bias in the dropout population (possibly due to COPD progression between visits likely resulting in increased risk of death/non-followup) might be discussed.

[Response] The reviewer makes a very valid point, which we failed to emphasize. Indeed, COPDGene recruited 10,725 people at baseline, but 47% have an unknown GOLD stage for a second visit. We have no tracking available (patient confidentiality), but we know that those 47% include 2,074 people who died and the rest were unreachable. The people who died might introduce some selection bias for all studies that use COPDGene with longitudinal measurements. Here, however, we are focusing on those that had normal spirometry (i.e., designated as GOLD 0) at the baseline visit. And this category has much lower mortality than the others. So, we expect that the selection bias due to death is small.

Furthermore, we note that the percentage of people that move out of GOLD 0 between visit 1 and visit 2 is about the same as between visit 2 and visit 3 (Suppl Fig S1B). So, selection due to mortality does not seem to have a major effect for our project. Nevertheless, we now list this issue in the Discussion as a potential limitation.

5. In the COPDGene Study Population subsection, it is stated that missing values were imputed with a k-nearest neighbours (k=5) algorithm. It might be clarified as to how imputation was performed for categorical data, with possible ties (e.g. 2-2-1 or 1-1-1-1-1, with a tie in the most common category)

[Response] We apologize to the reviewer for this confusion. We mention briefly in the methods that Gower's distance (in combination with SMOTE) was used to perform the imputation. The implementation we used is from the *themis* R package using the *smotenc()* function. In the documentation, it is stated that for categorical variables, the most common category among neighbors is chosen (i.e. statistical mode). In the event of ties, this package takes the same default approach as the base R *which.max()* function and returns the first-appearing value in the set of modes. We have added a sentence to the methods noting that we have used this specific package implementation for clarity.

6. The SCCOR dataset used for external validation appears to be of a similar (American) demographic, as the COPDGene development dataset. It might be considered to include external validation on additional populations/demographics if possible, to further support the generalizability of the model and results.

[Response] Unfortunately, there are no other COPD datasets with similar ascertainment criteria and such detailed phenotyping that are racially diverse. Both the other two major datasets available (SPIROMICS and ECLIPSE) have overrepresentation of non-hispanic white participants.

7. In the Causal Graph Algorithm subsection, it is stated that nested cross-validation was performed across ten α (graph sparsity parameter) values, maximizing AUROC. The nested cross-validation procedure (and data on which the AUROC was computed on) might be described in greater detail.

[Response] We apologize to the reviewer for the lack of clarity. We now describe the procedure in more detail in the supplemental methods.

8. It is then stated that AUROC was also maximized for logistic regression models using variables from a covering Markov blanked. Again, the nested cross-validation procedure might be described in greater detail. Moreover, would it have been more appropriate to jointly optimize the logistic regression model(s) together with the graph sparsity parameter α?

[Response] For the nested cross-validation, please see comments above as we believe they clarifies this point as well. Regarding the concurrent optimization of graph sparsity and prediction accuracy, this is indeed what we do, since we run a hyperparameter sweep and select the parameter value that optimizes AUROC curve. We understand that this needed more explanation, which we are now providing in the Supplemental Methods.

9. It is stated that 10-fold cross-validation was used to train the RF model(s). Was this equivalent to the "nested cross-validation" previously described, and if not, what was the reason for the discrepancy? The training methodology of the RF model, including details of hyperparameter optimization, might also be included - possibly in the supplementary material.

[Response] Yes. Both methods were trained on exactly the same subsets using the same cross-validation procedure. For the RF model specifically, there are multiple parameters that are optimized which we now clarify in the Supplemental Methods.

Also, to clarify, we use the term "nested cross-validation" to refer to the fact that in each round of cross-validation there is a variable selection step and a parameter optimization step. In this way, there is no leakage of information.

10. In the Linear graph-based models subsection, for the external validation results (Figure 5), the performance of the RF models does not appear to be included (unlike for internal validation results in Figure 4). This might be strongly considered.

[Response] The purpose of this figure was not necessarily to compare different predictive methods, but to show that our method could generalize to datasets beyond COPDGene. The reason we cannot include the RF model in this comparison is that many of the 131 variables in the COPDGene dataset (which the original RF model uses) are not included in the SCCOR dataset. We did run RF with the remaining 5 variables (Pi10 is not available in SCCOR) and the results are presented below. We founded it to be slightly inferior to our graph-based model, which has a SCCOR AUROC=0.89 (Fig. 5). This is not surprising as the RF model is being limited by the variable chosen by FCImax.

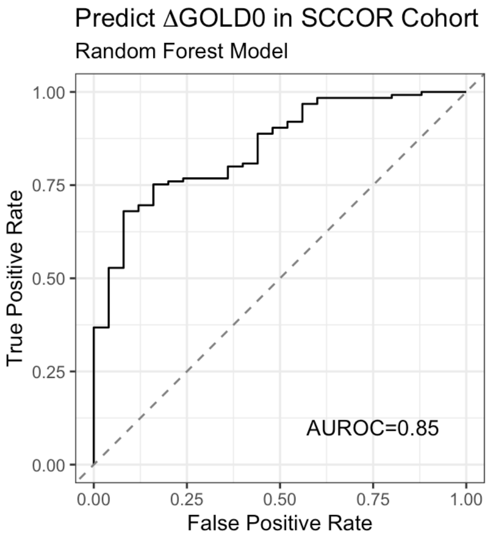

11. It is then stated that "There is a dependency between the training and internal validation datasets because the same individuals participate in both (although at different timepoints)". If so, it might be clarified as to why the training and internal validation datasets could not have been split at the patient level, such that all data from a patient would appear in either the training or internal validation split, and not both.

[Response] The reviewer brings up a good point that we were also concerned about. We initially performed a random split on the training and testing data because we did not want to introduce a selection bias. If we had reserved those that remained GOLD0 in the second visit, it might have resulted in unbalanced dataset of healthier vs at-risk subjects. The way we do it now, for the first part (training/testing) there is no bias. For the second part (internal validation) we run both the full validation dataset and the morse restricted internal validation that contains no individuals from the original training set (see Supplemental Figure S5). The results of these three runs and the external validation, in our opinion, are convincing of the model's generalizability.

12. It might be considered to briefly investigate LR/RF models trained with the top 6 variables by Shapley values (Figure 6), to further support the necessity of causal modelling over other feature importance methods.

[Response] The top variables as determined by the mean absolute Shapley value in Figure 6A were presented to help explain what variables contributed most to RF model predictions. In a way, these are the variables that RF chose as its top predictive features. Using these top 6 variables as a feature selection method to train additional models would be difficult to justify, especially since we do not know how many of the top variables help with possible non-linear effects. having said that, it is still interesting to observe that the models prioritize different variables for prediction than the graph models (e.g., LAA950), which we make note of in the Discussion.

To ensure these variables did not improve the model's predictive power, we ran a logistic regression using the top 6 variables from Figure 6A using the same testing/training split. We found the model resulted in an AUROC of 0.68 on the testing dataset (compared to 0.75 for the FCImax model and 0.70 for the full RF model).

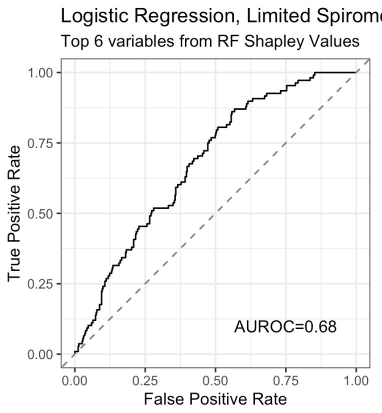

Reviewer #2:
ROC-AUC values do not have confidence intervals or p-values in the text. Those should be added.

[Response] This is a good point, and we apologize for the lack of clarification. In fact, we had included confidence interval shapes in Figure 4 for the training dataset (shaded area around the ROC curve lines). For the testing set, we cannot have confidence intervals since it is a single dataset. For ROC curve differences between the graph-based models and the RF models, there was no statistical significance, which is why we did not display them. This has been added to the figure 4 caption for clarification. Additionally, we include here a boxplot figure to illustrate the point.

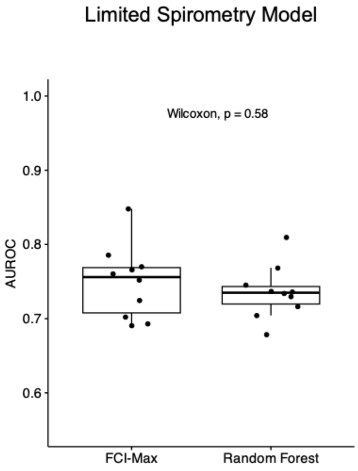

"This re-trained model had a small reduction in AUROC for the COPDGene testing dataset (from 0.75 to 0.73) but resulted in an AUROC of 0.89 when applied to the external SCCOR cohort (Figure 5). This indicates that our model's predictions are robust and generalize beyond COPDGene."

This is not only a much larger AUC than anything I have ever seen WRT prediction of decline in lung function, but is also a highly unusual performance increase from training to test datasets, almost too good as to be unbelievable unless there were some unique attributes in the SCCOR database. This should be addressed somewhere in the discussion.

[Response] We appreciate the reviewer's insights and past experience on prediction performance differences between training and validation datasets. We were pleased to see this increase and we believe that the differences in the cohort may explain the unexpected improvement in AUC. We had speculated in the Discussion:

"This improvement in model performance may be partially explained by the lower percentage of subjects leaving GOLD 0 status (20.3% vs 16.7%)."

In general, predicting "staying in GOLD 0" is easier due to more such examples, which is why we oversampled the "leaving GOLD 0" category during training (to avoid class imbalance).

In an attempt to further investigate this unusual performance increase, we looked at the data in both cohorts for the top predictor, FEF25-75%:

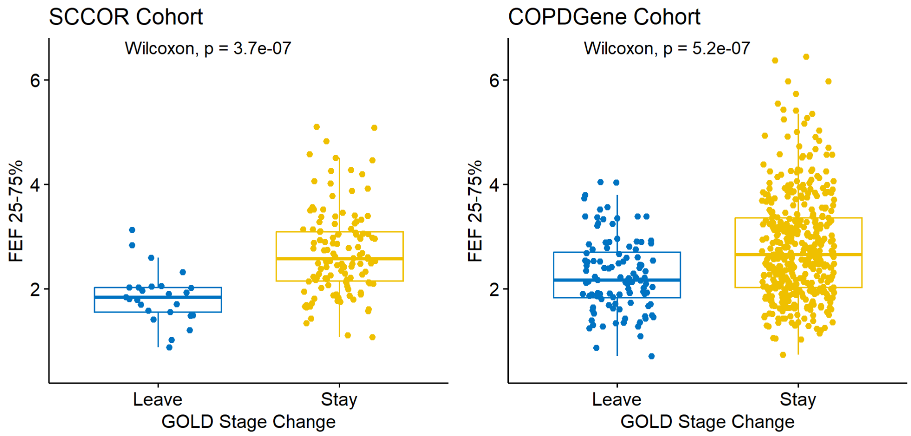

The p-values are similar; the average FEF25-75% for those that remain in GOLD 0 is also similar. However, the SCCOR participants that leave GOLD 0 have lower average FEF25-75% than the COPDGene participants; and better overall separation of and reduced variation within the two categories. We believe that this difference likely contributes to the increase performance we see for the SCCOR cohort. We added a paragraph in the section about linear graph modeling to highlight this.

I'd be curious to know why densitometry or lung volume was not considered as an important variable in the models. It's fine if this happens to be the case, but again it contradicts earlier research and thus there should be something in the discussion address why this happens in this dataset. Other research1 showed that expiratory lung volume and PRMfsad, both of which are available in the COPDGene dataset, were strong predictors of spirometric decline.

1Machine learning for screening of at-risk, mild and moderate COPD patients at risk of FEV1 decline: results from COPDGene and SPIROMICS, Wang et al.

[Response] We respectfully disagree with this reviewer that our study "contradicts earlier research". The paper mentioned has pre-removed what they considered as redundant variables and used different target variable (accelerated FEV1 decline) in different population (the PRM variable is associated with the GOLD 1-2 rapid decliners).

In our paper, we follow an agnostic approach for selecting variables. The densitometry variables, including density thresholds and parametric response methodology variables derived from the inspiratory and expiratory scan mapping, were all included as input. But the graphical models(through conditional independence tests) consider that their information content was superseded by the other selected variables ("limited spirometry" model). We consider this as an advantage of the causal graphs because they can look beyond correlation.

We would also like to point out that the "no spirometry" graph model includes densitometry variables like log inspiratory percentage below 950 HU (supplemental figure S4B) and the RF model uses "inspiratory % low attenuation area <950 HU (Right lobe)" as a strong predictor (Figure 6A).

So, overall, we believe that the paper mentioned by the reviewer (which we now discuss in our manuscript) makes convincing claims that variables derived from lung CT are important for predicting FEV1 decline, but we maintain the position that our paper does not contradict it, as we look on different outcomes, using somewhat different variables. Despite that, we find some of the same variables in our analyses.

This is my primary issue with the paper and what needs the most investigation/explanation for a revision: It would be good to plot the difference in FEV1 and FVC distributions between those who were deltaGOLD0=1 vs deltaGOLD0 in the training dataset. Although FEV1 and FVC were not considered in the limited spirometry model, FEF27-75 is highly correlated with FEV1/FVC, indicating that using FEF-25-75 is a proxy for a definition of GOLD 0. It may be that FEF25-75 is such a useful variable because it is highly correlated with FEV1/FVC and therefore is selecting subjects who are just on the border of GOLD0 - GOLD1 classification and therefore likely to convert via noise.

[Response] We thank the reviewer for this comment. This was a major concern for us as well, which is why we included the entire "no spirometry" model. Even without FEF25-75%, the "no spirometry" model can differentiate changes in GOLD 0 status using variables like Pi10, age, attack of wheezing/whistling in chest, and visual wall thickening on CT. Knowing that these variables carry direct information for future lung abnormalities over other measurements is still a novel finding that should be investigated.

But following reviewer's comment we further examined the "limited spirometry" model (which had excluded any variables directly derived from FEV1 or FVC). The reviewer is right that FEF25-75% is correlated with the FEV1/FVC ratio (R^2=0.717 in our training dataset). The main concern the reviewer has is that, because of this correlation, subjects on the GOLD0-GOLD1 border will become trivial to predict and this is what drives the good performance of the model. According to the reviewer, this transition can occur through (small) reduction to FEV1/FVC due to noise, thus might not be clinically significant. This argument has two extensions related to our "*limited spirometry*" model: (1) the model will poorly predict the GOLD0->PRISm transition, which is due to reduction in FEV1 %predicted (i.e., not affected by FVC); and (2) the initial FEV1/FVC of those transitioning should be around the threshold (i.e., 0.7). There are multiple lines of evidence indicating that this is not the case.

First, as we reported in the paper, FEF25-75% is an important predictor for lung abnormalities not only in the general model (combined GOLD1-4 and PRISm transitions). It remains significant for GOLD0->PRISm transition even in the separated models (see Table 2). This means that its predictive ability extends beyond its correlation to FEV1/FVC ratio. Second, we see that those that transition out of GOLD 0 can be quite far from the FEV1/FVC border (Y-axis, Fig. A below; now Suppl. Fig. S8A) and the model still predicts them correctly (even when their initial FEV1/FVC value is ~0.85; see Fig. B below). In fact, the mistakes the model makes (i.e., the subjects incorrectly predicted as staying in GOLD 0) are for the most part in the periphery of those leaving GOLD 0, in both directions (FEV1/FVC and FEV1 %predicted; Fig. B, dotted line).

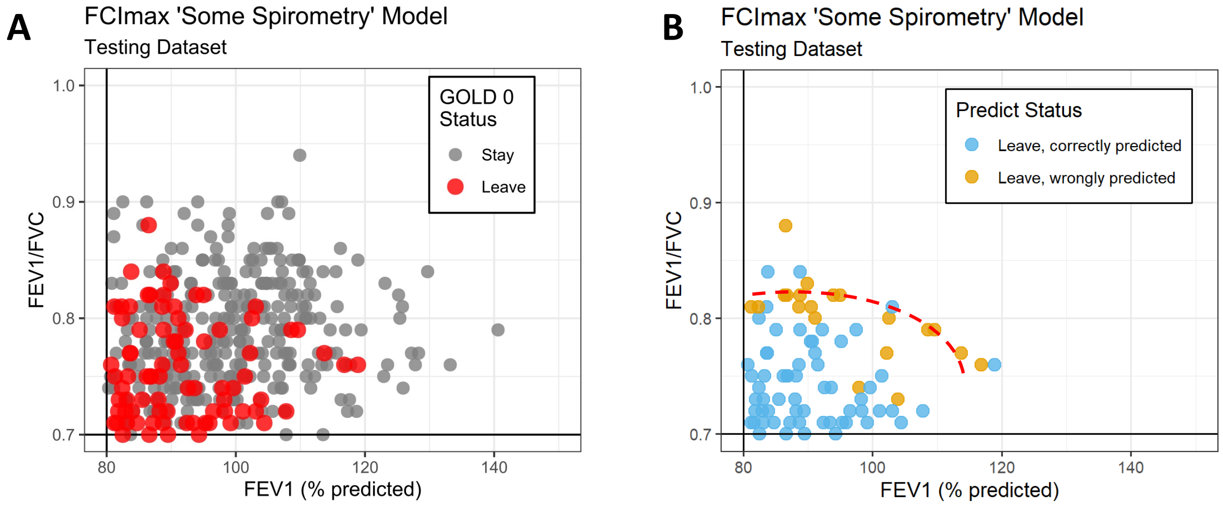

Finally, we run a predictive model with only FEV1/FVC as predictor and we found it to be inferior to our model (average AUROC= 0.673, compared to 0.75 for our model; see ROC curve below).

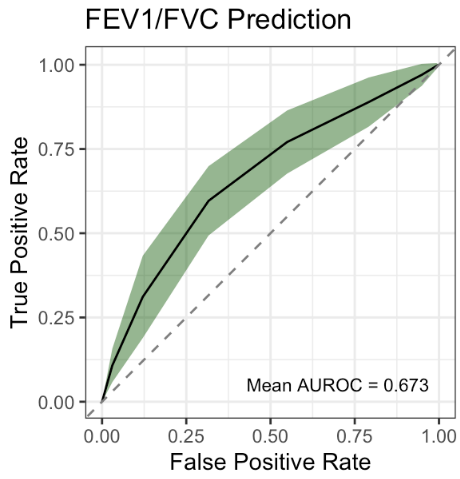

These evidence, taken together, suggest that although one aspect of the FEF25-75% predictive capacity might be due to its selection of subjects on the border of GOLD0->GOLD1 conversion, other aspects of this metric that are related to early midflow impairment and is not captured within FVC, may contribute to its more general predictive capacity. The general importance of FEF25-75% was missed by other studies (including the one mentioned by the reviewer) because they preemptively excluded FEF25-75% before their analyses.

We have now added a separate section in the paper to discuss the value of FEF25-75% and its correlation to FEV1/FVC in our predictive model.

This is a problem with predicting change in GOLD status rather than either regression of FEV1 or classification of significant change in FEV1.

[Response] This is a good point. Let's clarify. Predicting future FEV1 (as change or as binary variable for rapid FEV1 decline) is very useful when the objective is to study progression mainly within COPD patients (from GOLD 1 to other GOLD categories). This is what other studies (like the one the reviewer references above) have done. However, we are interested on factors affecting the development of *any* lung functional abnormalities from spirometrically normal (GOLD 0) subjects. In this case, we need to be able to model changes in both FEV1 (%predicted) and FEV1/FVC ratio. This is the focus of our study. And this is what we think that the major contribution of FEF25-75% is since it appears to carry information for changes in both directions (see Table 2).

The Markov blanket seems to indicate that deltaGOLD0 causes change in FEF, not the other way around. This seems like a major problem with the graph model because the hypothesis is that FEF would predict GOLD progression, correct?

[Response] We apologize for this misconception. The arrow from ΔGOLD0 to FEF in our original Fig. 2 had a round end in ΔGOLD0, meaning that a latent confounder may be present. We understand that this could be confusing sometimes. To avoid this confusion, we modified the diagram in Fig. 2 and designated with bidirectional arrow every instance where there was a possibility for a latent confounder affecting the two connected variables.

Which Pi10 was used? VIDA, Thirona, or something else?

[Response] This is Thirona.

"Unmeasured inflammation may be the latent confounder between Pi10 and ΔGOLD0 as our model predicts". Doesn't COPDGene have some C-RP measurements? Could you look at those too, at least in a subset?

[Response] Our focus for this paper was to look only on the usual variables that can be collected on baseline, like demographics, spirometry, and radiological variables. This is why we did not initially include blood-derived measurements (gene expression, proteomics, metabolomics, SNPs, etc). We expect inflammatory biomarkers like C-RP (and others: SP-D, sRAGE, etc. (Zemans et al., 2017)) to play a role in explaining the connection between Pi10 and ΔGOLD0, but the comment the reviewer mentions above is just a suggestion, based on previous reports. Besides, it is not clear to us that such a claim can be proven, which is why we simply mention it in the Discussion as a possible explanation of the connection we found. In future work, we plan to extend this study to include omics variables, but if the reviewer feels strongly about this comment, we can remove it from the Discussion.

Reviewer #3:
The manuscript "Causal Graph Modelling Identifies Six Factors Linked to Incident COPD" by Gregg et al. is certainly an important advance in the understanding of chronic obstructive pulmonary disease (COPD). In essence, this research identifies relevant variables that influence the decline in lung function. This study

includes clinical, genetic, and radiological features to identify the predictors of lung function decline. Causal graph modelling techniques were used on a large cohort of 2,643 subjects with a history of smoking but normal spirometry. The analysis identified six key variables that were associated with lung function abnormalities. Among these variables, factors such as "FEF25-75%" and "airway wall thickness" were found to be highly predictive of COPD progression. In addition, the use of predictive models using logistic regression and random forest algorithms added value to the results. An area under the receiver operating characteristic curve (AUROC) value of 0.75 demonstrated the robustness of the model in correctly classifying patients who experience a decline in lung function from those who maintain stable function over a 5-year period. External validation in an independent cohort strengthens the generalisability of the results and provides reasonable support for the reproducibility of the identified predictors in different populations. Importantly, the agnostic approach of this study contributes to the understanding of the heterogeneity of COPD by delineating distinct trajectories of disease progression. The findings pave the way for personalised interventions tailored to specific COPD subtypes. Such insights hold great promise for improving risk stratification, early detection and targeted therapeutic interventions, ultimately improving the management and prognosis of COPD patients. In conclusion, I think the manuscript is well written, well organised and the results seem to be very solid, of great interest to PLoS
Medicine readers and very worthy of publication as is.

[Response] We are grateful to this reviewer for the thorough review of our paper and their appreciation of our work.

References

Probst, P., Wright, M. N., & Boulesteix, A. L. (2019). Hyperparameters and tuning strategies for random forest. *Wiley Interdisciplinary Reviews: Data Mining and Knowledge Discovery*, *9*(3), e1301. https://doi.org/10.1002/WIDM.1301

Zemans, R. L., Jacobson, S., Keene, J., Kechris, K., Miller, B. E., Tal-Singer, R., & Bowler, R. P. (2017). Multiple biomarkers predict disease severity, progression and mortality in COPD. *Respiratory Research*, *18*(1), 1–10. https://doi.org/10.1186/S12931-017-0597-7/TABLES/6
