# Supplementary material for: Identification of factors directly linked to incident chronic obstructive pulmonary disease: A causal graph modeling study
Source: PLoS Med. 2024 Aug 13;21(8):e1004444. doi: 10.1371/journal.pmed.1004444 (PMC11349214; doi:10.1371/journal.pmed.1004444)
Supplement: Attachment — Submitted filename: 2_COPD_onset.Response_R3.draft20240710.pdf [file pmed.1004444.s014.pdf]

POINT-BY-POINT RESPONSE TO EDITORIAL COMMENTS

TITLE

Please revise your title according to PLOS Medicine's style. Your title must be nondeclarative and not a question. It should begin with main concept if possible. "Effect of" should be used only if causality can be inferred, i.e., for an RCT. Please place the study design ("A randomized controlled trial," "A retrospective study," "A modelling study," etc.) in the subtitle (ie, after a colon). We also suggest that you spell out "COPD".

[Response] We revised the title to "*Causal Graph Modeling Identifies Six Factors Linked to Incident Chronic Obstructive Pulmonary Disease: a retrospective study*".

FINANCIAL DISCLOSURE

Please state whether any sponsors or funders (other than the named authors) played any role in study design, data collection and analysis, the decision to publish, or preparation of the manuscript. If they had no role in the research, include this sentence: "The funders had no role in study design, data collection and analysis, decision to publish, or preparation of the manuscript."

[Response] We added the Financial Disclosure in the cover page.

DATA AVAILABILITY

The Data Availability Statement (DAS) requires revision. For each data source used in your study:

a) If the data are freely or publicly available, note this and state the location of the data: within the paper, in Supporting Information files, or in a public repository (include the DOI or accession number).

[Response] This is the case for the COPDGene data, which are available through dbGAP. We have listed the dbGAP accession number (phs000179.v6.p2) in the Data Availability portion (lines 512-514).

b) If the data are owned by a third party but freely available upon request, please note this and state the owner of the data set and contact information for data requests (web or email address). Note that a study author cannot be the contact person for the data.

c) If the data are not freely available, please describe briefly the ethical, legal, or contractual restriction that prevents you from sharing it. Please also include an appropriate contact (web or email address) for inquiries (again, this cannot be a study author).

[Response] We added the relevant text and contact person. Briefly, the SCCOR cohort consent form does not allow for the data to be publicly shared, but the clinical data used in the validation of the model can be shared upon request.

CODE SHARING

We expect all researchers with submissions to PLOS in which author-generated code underpins the findings in the manuscript to make all author-generated code available without restrictions upon publication of the work. In cases where code is central to the manuscript, we may require the code to be made available as a

condition of publication. Authors are responsible for ensuring that the code is reusable and well documented. Please make any custom code available, either as part of your data deposition or as a supplementary file. Please add a sentence to your data availability statement regarding any code used in the study, e.g. "The code used in the analysis is available from Github [URL] and archived in Zenodo [DOI link]" Please review our guidelines at https://journals.plos.org/plosmedicine/s/materials-software-and-code-sharing and ensure that your code is shared in a way that follows best practice and facilitates reproducibility and reuse. Because Github depositions can be readily changed or deleted, we encourage you to make a permanent DOI'd copy (e.g. in Zenodo) and provide the URL.

[Response] We are big proponents of open science, and we always make our new methods available to the community either through our GitHub repository or through our web server. However, in this case, we did not create new software. The code we generated consists of a set of simple scripts, using off-the-self methods to parse, and analyze the data. In the paper, we detailed the software we used and the methods and thresholds we used for the analysis, if they differ form the default values, so other people with access to the data can easily replicate our results. However, since the data are available to the general public through dbGAP, and we are not allowed to put them in a public repository (e.g., GitHub), our in-house scripts will have no value to the community. As a result, we did not add a Code Sharing section, because it is not applicable in our case.

ABSTRACT

1) l.23, please change to: This study aimed..."

[Response] Done.

2) l.25ff: Please define abbreviations used, such as 'FEF25-75%', 'Pi10', 'AUROC' and 'FEV1'.

[Response] Done.

3) l.25ff: Please replace "subject" with participant, patient, individual, or person. Please revise throughout the Abstract and the main text.

[Response] We have now replaced the word "subject" with "participant" or "individual", depending on the context, throughout the text.

4) l.25ff: Please revise the Abstract with regard to the appropriate tense. Methods and Results should be presented in the past tense.

[Response] Done.

5) l.25ff: Please provide details about the individuals enrolled in the study, such as age, sex, race/ethnicity. Please provide the dates of enrollment (for the COPDGene study). Please also do so for the cohort used for external validation.

[Response] Done.

6) ll.29-30: Since the six variables identified in your study are part of the main finding, we suggest listing all of them in the Abstract. We also suggest mentioning that you aimed to determine if the variables could predict individuals leaving GOLD 0 status (normal spirometry according to Global Initiative for Obstructive Lung Disease (GOLD) criteria).

[Response] Done.

7) l.31: Please write ‘yr’ in full.

[Response] Done.

8) l.31: Please provide the numerical results for the Random Forest predictors.

[Response] Done.

9) l.33: Please define ‘SCORR’ and include the results (i.e., AUROC) for the external validation.

[Response] Done.

10) In the last sentence of the Abstract Methods and Findings section, please describe the main limitation(s) of the study's methodology.

[Response] Done.

AUTHOR SUMMARY

1) Please revise the Author Summary regarding the appropriate tense. For example, ‘Airway wall thickness indicated...’ and/or ‘Methods like graphical models could be used…’.

[Response] Done.

2) l.41: Please define ‘COPD at first use.

[Response] Done.

3) l.46ff: Please define all abbreviations the first time you use them or write them out in full.

[Response] Done.

INTRODUCTION

l.92, please change to: “In this study, we leveraged…” and revise the following sentences accordingly (i.e. the use of past tense).

[Response] Done.

METHODS AND RESULTS

We feel that the Results section could be improved in terms of clarity and readability. It is important to guide the reader through the results. In the current format, the connection and introduction of paragraphs sometimes seems a bit disruptive. Please keep in mind that the manuscript should be written in a way that is accessible to a broad audience and to readers who are not familiar with the topic.

[Response] We did our best to clarify certain points for the non-pulmonologists. This was done using rephrasing or adding more text to explain the background.

1) l.105: Please define ‘CT’ at first use.

[Response] Done.

2) l.112: We suggest not abbreviating the word "years" with "yrs" and using the full word instead. Please revise throughout the main text.

[Response] Done.

3) Please include the following statement in the Methods section (We noted it was only included in the ‘SUPPLEMENTAL MATERIALS and METHODS’) and ensure to provide the completed checklist as a Supporting Information file: “This study is reported as per the Transparent Reporting of a Multivariable Prediction Model for Individual Prognosis Or Diagnosis (TRIPOD) statement (S9 Checklist).”

[Response] We added this statement at the end of the Materials and Methods section.

4) ll.207-208: We suggest replacing the subheading with a more general statement, e.g. ‘Variables linked to 5-year ΔGOLD0 progression’.

[Response] Done.

5) l.209: As you begin to describe the results here, we feel that it may be confusing to the reader to begin the paragraph with "After identifying..." as it suggests that another results section must have preceded it. Please rephrase.

[Response] We have now added an introductory sentence and rephrased.

6) ll.279-280, please revise for clarity.

[Response] We revised this section. We now provide more information to explain why the correlation between FEF25-75 and FEV1/FVC might create “information leakage”. So, the testing (and disproval) of this hypothesis becomes clearer.

7) ll.306-307: We suggest replacing the subheading with a more general statement, e.g. ‘Contribution of demographics and SNPs to the prediction of ΔGOLD0 in the random forest model’.

[Response] Done. We now use the suggested subheader.

8) l.309: How was race/ethnicity defined and by whom? We suggest using the term ‘ethnicity’ instead of ‘race’.

[Response] Race was self-reported during the initial interviews. Actually, the COPDGene questionnaire had two questions: one for race and one for ethnicity. We used the variable representing the answers that were given to the former question, in agreement with all the other studies that have analyzed COPDGene data. For example, please see our recent publication Regan et al, 2024, Am J Resp Crit Care Med, “*Early Evidence of COPD Obscured by Race-Specific Prediction Equations*”. Given these, we believe it is more appropriate to use the self-reported “race” instead of “ethnicity”, first because this reflects the answer the participants gave; and second, to be consistent with previous publications and avoid confusing the people that follow the COPD literature.

9) l.311, please change to: "…of 65-70 years."

[Response] Done.

DISCUSSION

1) Please remove any subheadings from the Discussion.

[Response] Done.

2) When discussing methods and results, please use the past tense.

[Response] We believe we accomplished this.

3) We suggest more explicit discussion of the limited clinical utility of the model.

[Response] In the previously labeled as "Limitations" section of the Discussion, we have now added the following:
"Being able to predict who is going to progress is of high value for people at risk of developing COPD. However, our model's clinical utility is restricted by the limited racial diversity in the training cohorts we used and the fact that all participants were older with extensive history of smoking (>10 pack-years), which makes them high-risk. This impacts current clinical utility until we evaluate the model in diverse cohorts and cohorts with lower and no smoking histories. Another limitation is that Pi10, which is an important contributor to the predictive models, can only be obtained through CT scans. This limits the models' applicability in resource limited settings."

TABLES AND FIGURES

1) Table 2: Please ensure that you define all abbreviations used in the table, such as 'FEV1/FVC' or 'AUROC'. Please add a unit for age, e.g. 'years'.

[Response] Done.

2) Figure 1/2/3/4/5/6: Please ensure that you define all abbreviations used in the figures, such as 'FEV1/FVC' or 'AUROC'.

[Response] Done.

3) Figure 3: Please change to 'Markov Blanket' (l.622).

[Response] Done.

4) Figure 4: What does the shaded area in graphs (A) and (D) indicate?

[Response] The shaded area designates the confidence interval for the training set, where we do cross-validation (and thus we can calculate a confidence interval). It is meant to show that there is no difference in performance between the graph-based linear model and the (non-linear) random forest. We now added it in the caption.

5) Supplemental Figure S3: Please report exact p-values.

[Response] Done.

6) Supplemental Figures: Please ensure that you define all abbreviations used in the figures, such as ‘FEV1/FVC’ or ‘AUROC’.

[Response] Done.

REFERENCES

1) Where website addresses are cited, when specifying the date of access, please use the word “accessed” instead of “cited” (e.g. [accessed: 10/04/2024]).

[Response] Done.

2) Please ensure that journal name abbreviations match those found in the National Center for Biotechnology Information (NCBI) databases (https://www.ncbi.nlm.nih.gov/nlmcatalog/journals) and are appropriately formatted and capitalised. For example, “New England Journal of Medicine” in reference [3] should be “N Engl J Med”.

[Response] Done.

SOCIAL MEDIA

[Response] Done.

To help us extend the reach of your research, please provide any X (formerly known as Twitter) handle(s) that would be appropriate to tag, including your own, your co-authors’, your institution, funder, or lab. Please enter in the submission form any handles you wish to be included when we post about this paper.

[Response] Done. (see also below)

Authors:
@TakisBenosLab
@FrankS1956

Funders:
@nih_nhlbi
@NLM_NIH

Institutions:
@UFPHHP
@UFMedicine
@UPMC
